# Hydration Status in Geriatric Patients—Subjective Impression or Objective Parameter? The Hydr-Age-Study

**DOI:** 10.3390/nu17193129

**Published:** 2025-09-30

**Authors:** Linda Deissler, Matthias Janneck, Rainer Wirth, Alexander Fierenz, Ulrich Thiem, Alexander Rösler

**Affiliations:** 1 Medical Faculty, University of Hamburg, Martinistrasse 52, 20251 Hamburg, Germany; 2 Department of General Internal Medicine and Nephrology, Albertinen Hospital, 22457 Hamburg, Germany; 3 Department of Geriatric Medicine, Marien Hospital Herne–University Hospital, Ruhr-University Bochum, 44625 Herne, Germany; 4 Institute for Medical Biometry and Epidemiology, University of Hamburg, Martinistrasse 52, 20251 Hamburg, Germany; 5 Department of Geriatrics, Medical School, University of Bielefeld, 33615 Bielefeld, Germany; 6 Agaplesion Bethesda Hospital Bergedorf, 21029 Hamburg, Germany

**Keywords:** hydration, aged, diagnostic accuracy, axillary dryness, ultrasonography

## Abstract

**Background/Objectives:** Assessing the hydration status (HS) in geriatric patients remains challenging due to multimorbidity, polypharmacy, and cognitive impairment. Common indicators like reduced skin turgor and dry mucous membranes are unreliable. The Hydr-Age-Study is a prospective observational pilot study with a post hoc analysis to evaluate the diagnostic accuracy of clinical, laboratory, and instrumental methods to assess HS in hospitalised older adults. **Methods**: Upon admission, patients underwent an assessment including their medical history, a clinical evaluation, laboratory tests, ultrasound examination, and bioimpedance analysis. These data were collected and independently reviewed by two experts who diagnosed each patient’s current HS. This diagnosis served as the clinical reference standard for evaluating the diagnostic accuracy of each method. **Results**: Twenty-six methods were examined, of which four achieved an AUC > 0.8. Axillary dryness showed a high diagnostic accuracy for hypohydration (AUC = 0.854), with a sensitivity of 83.3% and a specificity of 82.8%. Inferior vena cava (IVC) ultrasound effectively detected both hypo- and hyperhydration. A subxiphoid IVC diameter ≤ 1.95 cm identified hypohydration with 90.9% sensitivity and 50.6% specificity. For hyperhydration, a diameter of ≥2.15 cm provided strong diagnostic performance in both subxiphoid and transcostal views. **Conclusions**: Axillary dryness and IVC sonography demonstrated the highest diagnostic accuracy. No other methods exceeded an AUC of 0.80. In the absence of a gold standard, a structured clinical consensus provides a feasible and reproducible approach to establish a clinical reference standard. These findings may contribute to the development of a standardised assessment protocol in geriatric medicine.

## 1. Introduction

Assessing the hydration status (HS) of geriatric patients is crucial for their medical care. Shifts in body water and electrolyte balance are among the most frequently observed diagnoses in older adults [1,2].

The academic distinction between different states of hydration (dehydration vs. hyperhydration and hypovolemia vs. hypervolemia), as well as their physiological regulation is well known in young, healthy adults. However, volume and hydration status and their regulation do overlap, especially in older patients. Water and sodium homeostasis are governed by shared physiological mechanisms, including renal function, thirst perception, and hormonal regulation (notably the renin–angiotensin–aldosterone system and vasopressin) [3,4,5]. In older adults, age-related changes such as isosthenuria, diminished thirst sensation, and altered hormonal responses impair both volume and hydration regulation, increasing susceptibility to both dehydration (water deficit) and dysnatremias (hyponatremia and hypernatremia) [3,4,5,6].

Clinical manifestations of dehydration in older patients often reflect disturbances in both extracellular fluid volume and osmolality. The distinction between a pure water deficit (low-intake/hypertonic dehydration) and combined water and sodium loss (isotonic dehydration) is frequently blurred due to overlapping aetiologies and impaired compensatory mechanisms, such as compromised thirst and renal adaptation [4,7,8].

Therefore, in the present study, hydration and volume status are considered jointly under the umbrella term “hydration status” (HS). Hypohydration represents a state of fluid and/or volume deficiency and hyperhydration a state of fluid and/or volume overload.

Hyperhydration is a clinical diagnosis based on symptoms such as peripheral oedema, dyspnoea, and weight gain, which examiners do not find difficult to identify. Yet objective and reproducible methods are lacking, especially in the older population. For instance, there is no interdisciplinary threshold for detecting hyperhydration via ultrasound of the inferior vena cava (IVC).

Diagnosing hypohydration is more challenging. Traditional signs, such as reduced skin turgor and dry mucous membranes, have been shown to be unreliable in older adults [7,9,10]. Various clinical, laboratory, and instrumental methods have been investigated in the past for their diagnostic utility, mostly for identifying low-intake dehydration. While laboratory parameters like serum sodium and osmolality are part of the standard diagnostic workup to assess the HS in the older population, other methods, such as axillary dryness, laboratory measures (e.g., blood urea nitrogen to creatinine ratio (BUN/Cr)), ultrasound of the IVC, and bioimpedance analysis (BIA) have also been discussed [7,9,10,11,12,13,14,15].

The present preliminary pilot study aimed to evaluate the diagnostic accuracy of clinical, laboratory, and bedside instrumental methods for determining the overall HS in geriatric patients. Secondary outcomes included the feasibility of the clinical reference standard in diagnosing the *overall* HS (described below), as well as the prevalence and characteristics of the identified HS categories. For this purpose, we planned this prospective observational pilot study with a post hoc evaluation of the diagnostic quality of the used methods to assess the HS.

## 2. Materials and Methods

### 2.1. The Missing Gold Standard

**Figure 1:**
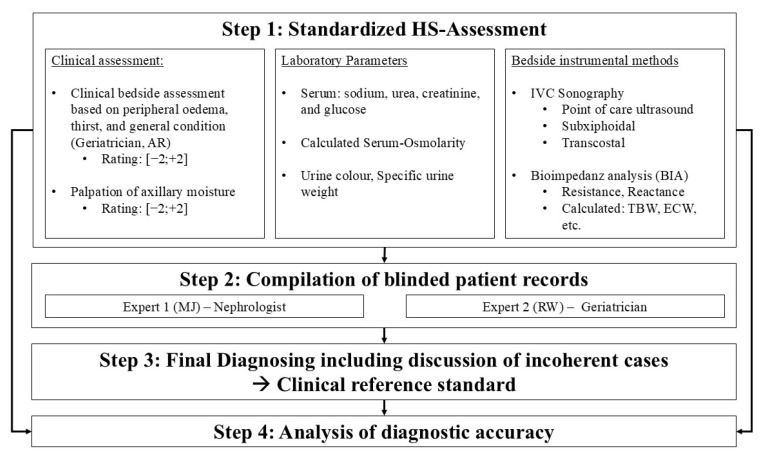
Diagnostic procedure for the evaluation of the overall hydration status.

To date, there is no universally accepted gold standard for assessing HS in geriatric patients [13,16]. In present literature, reproducibility and comparability are impaired by the utilisation of diverse and at times poorly described reference standards [7]. Furthermore, the assessment of a global HS has not been proposed before. Utilising standard references for only low-intake dehydration or hypovolemia for this approach is not a rational course of action. The STARD guidelines for reporting diagnostic accuracy define a clinical reference standard as the best available method to determine the presence of the target condition [17]. In evaluating the overall HS in geriatric patients, no single test by itself appears to be sufficient to discriminate between all possible conditions [7]. Therefore, the most reliable diagnostic approach appears to be an informed clinical assessment that integrates all relevant information. Recognizing this lack of a diagnostic gold standard, the following study protocol was designed. Figure 1 additionally displays our diagnostic procedure.

### 2.2. Study Design and Patients

This study was conducted in an acute care geriatric ward at Agaplesion Bethesda Hospital Bergedorf in Hamburg, Germany, from November 2022 to August 2023. Due to the explorative nature of investigating the overall hydration status, the sample size was planned, in agreement with the statistician, based on feasibility and expected drop-out. Patients admitted to this unit were screened for eligibility within the first 48 h of admission. The inclusion and exclusion criteria are provided in Supplementary Materials Table S1. The study protocol was approved by the local ethics committee on 14 January 2022 (Reference number: 2021-200275-BO-ff). The exclusion criterion of a Mini Mental Status Examination (MMSE) < 23 points was chosen to ensure, that all patients were capable to fully comprehend the study protocol. All patients provided written informed consent prior to participation.

### 2.3. Baseline Procedures

Blood and urine samples were routinely collected as part of the admission examination. Serum osmolarity was calculated according to the formula by Khajuria and Krahn [18]. BUN was calculated based on serum urea using the following formula: $BUN\left[\frac{mg}{dL}\right]=S\text{\_}Urea\;[\frac{mg}{dL}]\times 0.46$. Urine colour was rated in a 10 mL monovette against a white background using the urine colour chart by Armstrong [19]. Each patient underwent IVC sonography in both subxiphoid and transcostal views (General Electric, LOGIC P9 (GE HealthCare, Chicago, IL, USA), 3–5 MHz). BIA was conducted using AKERN BIA 101 (Akern SRL, Pisa, Italy). IVC sonography and BIA measurements were performed bedside in a supine position.

Anthropometric measurements were extracted from each patients’ medical records. Axillary moisture and a spontaneous clinical rating of each patient’s HS were assessed by an experienced physician (AR, Geriatrician). Axillary moisture was rated on a scale from −2 = very dry to +2 = very moist. The hydration status was rated from −2 = severely dehydrated to +2 severely hyperhydrated. The physician (AR) was blinded to the patients’ cause of admission, medical history, current medication, and all other assessments described above. All examinations were performed on the same day as the routine admission examination.

### 2.4. Clinical Reference Standard

In the absence of a diagnostic gold standard, the final clinical reference standard was determined by two independent experts from different locations, both with extensive experience in geriatrics and nephrology (RW and MJ). For each patient, a chart was prepared including the results of all assessments described above. The spontaneous clinical rating, as well as clinically relevant information such as the cause of admission, medical history, and current medications were also included. After independent review, the experts assigned each patient to one of three categories: hypohydration, euhydration, and hyperhydration. In cases of disagreement, cases were discussed in a virtual consensus meeting, where additional information (e.g., blood glucose levels) was provided. The resulting consensus diagnosis was used as the clinical reference standard for analysing the diagnostic accuracy of each method.

### 2.5. Statistical Analysis

Statistical analyses were performed using IBM SPSS (IBM SPSS v.29.0, IBM Corp., Armonk, NY, USA). The primary endpoint was to assess the diagnostic accuracy of the performed measures in classifying HS as hypohydration, euhydration, and hyperhydration. The secondary aim was to evaluate the utility of the clinical reference standard in subsequent statistical analyses. Inter-rater reliability between the two physicians was calculated using Cohen’s kappa and interpreted according to Landis and Koch [20]. Continuous data were presented as mean ± standard deviation (SD) or median ± interquartile range (IQR), as appropriate. Categorical data were summarised as absolute and relative frequencies. Due to small group sizes, group comparisons were performed using the Kruskal–Wallis test. In case of significance, pairwise post hoc comparisons were performed using the Mann–Whitney U test. Categorical variables (e.g., gender) were compared using the Pearson chi-square test.

To assess diagnostic accuracy, receiver operating characteristics (ROC) curves were calculated for each individual method of detecting the HS. Hypohydration was evaluated against the non-hypohydrated categories (euhydration and hyperhydration). Hyperhydration was evaluated against the non-hyperhydrated categories (euhydration and hypohydration). The area under the curve (AUC) was used as a global measure of diagnostic accuracy. Only methods with an AUC greater than 0.8 were considered for further analysis. For continuous variables, optimal cut-off values were identified using the Youden index [21]. Diagnostic performance measures included sensitivity, specificity, positive predictive value (PPV), negative predictive value (NPV), and the positive and negative likelihood ratios (LR+, LR−). The 95% confidence intervals (CI) were determined for all diagnostic parameters using the Wilson method (for proportions) or score method (for likelihood ratios) [22].

## 3. Results

### 3.1. Participant Characteristics and Group Differences

In total 103 patients were primarily included in the study, of which two had to be excluded, since the planed blood sampling could not be performed within 48 h of their admission.

A total of 101 geriatric patients were included in our analysis, 58.4% of whom were female. The average age was 80.1 ± 6.97 years. Further characteristics and group differences categorised by final diagnosis are shown in Table 1. Twelve patients were diagnosed as hypohydrated, 69 as euhydrated, and 20 as hyperhydrated.

Gender distribution varied between hydration groups, with the highest proportion of women (83.3%) in the hypohydrated group. There were relevant differences between the groups in terms of body weight, height, and body mass index (BMI). On average, the hyperhydrated group had the highest body weight and BMI. To analyse these differences in more detail, pairwise comparisons were performed using the Mann–Whitney U test.

Hyperhydrated patients had a relevantly higher body weight (*p* < 0.001) and BMI (*p* < 0.001) than hypohydrated and euhydrated patients, while height did not differ between the hyperhydrated and euhydrated groups (*p* = 0.494). There was a relevant height difference between the hypohydrated and hyperhydrated groups (*p* = 0.003). Age (*p* = 0.594) and cognitive ability (MMSE; *p* = 0.594) did not differ substantially.

### 3.2. Laboratory Measurements

Results from all laboratory measures are summarised in Table 2. The analysis of group differences was performed using the Kruskal–Wallis test. None of the serum parameters, including serum osmolarity and sodium or the urine parameters differed between the hypohydrated, euhydrated, and hyperhydrated states.

### 3.3. Inter-Rater Reliability

To assess the agreement between the two independent raters (MJ and RW), inter-rater reliability was calculated. Calculated agreement was Κ = 0.687 (Standard error (SE) = 0.074), which is considered as “substantial agreement” according to Landis and Koch [20].

The agreement between the initial spontaneous bedside rating of the HS of each patient (AR) and the final diagnosis was also examined, resulting in a kappa value of Κ = 0.728 (SE = 0.067; *p* < 0.001), also indicating a substantial agreement.

### 3.4. ROC Analysis and Diagnostic Accuracy in Assessing the HS

In total, 26 different parameters and methods were evaluated for their diagnostic accuracy in detecting either hyper- or hypohydration. All AUC results are summarised in Supplementary Materials Table S2.

#### 3.4.1. Detecting Hypohydration

Two methods achieved an AUC greater than 0.8: Axillary dryness (AUC = 0.854) and sonographic measurement of the maximal diameter of the IVC in subxiphoid view (AUC = 0.834).

- Axillary Dryness

Axillary moisture was rated for each patient on a scale from −2 to +2 (−2 indicating a “very dry” skin in the axilla and +2 indicating “very moist” skin). Searching for a simple and easy-to-perform test to detect hypohydration, the diagnostic accuracy was calculated for the presence of a “dry axilla”, including all ratings below zero. This method showed a sensitivity of 83.3% (95% CI: 55.2; 95.3%) and specificity of 82.8% (95% CI: 73.5; 89.3%). Further markers of diagnostic accuracy are shown in Table 3.

- Inferior vena cava sonography

Only ultrasound in subxiphoid angle achieved an AUC greater than 0.8. Using the Youden index, a cut-off value of ≤1.95 cm was identified (Jouden Index = 0.506). The subsequent calculation of diagnostic accuracy showed a sensitivity of 90.9% (95% CI: 62.3; 98.4%) and a specificity of 50.6% (95% CI: 39.8; 61.4%). Further markers of diagnostic accuracy are shown in Table 3. For a better comparability, we also displayed markers of diagnostic accuracy of different cut-off values, which both resulted in a lower Jouden Index (cut-off < 1.55 cm = 0.421; cut-off < 2.15 cm = 0.367).

#### 3.4.2. Detecting Hyperhydration

In this analysis, four different methods achieved an AUC greater than 0.8: BMI (AUC = 0.802), body weight (AUC = 0.802), and sonographic measurement of the maximum diameter of the IVC in both subxiphoid (AUC = 0.849) and transcostal views (AUC = 0.845). In our analysis only IVC ultrasound was further tested for its diagnostic accuracy.

- Inferior vena cava sonography

Measuring the IVC in the subxiphoid view resulted in an AUC of 0.849. A cut-off value of ≥2.15 cm was determined using the Youden index, resulting in a sensitivity of 73.7% (95% CI: 51.2; 88.2%) and a specificity of 78.9% (95% CI: 68.0; 86.8%).

Ultrasound in transcostal view to detect hyperhydration resulted in an AUC = 0.845. This method showed a sensitivity of 88.9% (95% CI: 56.5; 98.0%) and a specificity of 71.4% (95% CI: 54.9; 83.7%). Further markers of diagnostic accuracy are shown in Table 4.

As described above, we also included different cut-offs for the IVC diameter in our analysis. The cut-off value of ≥2.15 cm resulted in a Jouden Index of 0.648 for the transcostal and 0.648 for the subxiphoid angle. All other cut-offs did not reach a comparable Index (Jouden Index: subxiphoid angle: cut-off ≥ 2.50 cm = 0.452; cut-off ≥ 1.55 cm = 0.379; transcostal angle: ≥2.50 cm = 0.375; ≥1.55 cm = 0.276).

## 4. Discussion

The aim of this study was to evaluate the diagnostic accuracy of various methods for assessing the overall HS in geriatric inpatients. We used a clinical reference standard (Step 3, Figure 1) that combined the judgement of two independent physicians who had full access to all clinical and diagnostic information to assess the HS but were not part of the initial HS assessment (Step 1, Figure 1). Spontaneous ratings based on clinical signs alone do not appear to be sufficient, since traditionally used signs and symptoms have not demonstrated sufficient diagnostic accuracy [7]. The substantial agreement between the two raters in our protocol suggests that it is feasible to reliably evaluate the overall hydration status following our procedure [23,24]. This finding of our preliminary pilot study has to be further validated in follow-up study protocols.

### 4.1. Hypohydration

Surprisingly, only 12% of the participants were diagnosed with hypohydration. The high rate of low-intake dehydration reported by nursing homes and from emergency rooms might not apply anymore to patients, once they arrive at our geriatric care ward after receiving previous treatment (e.g., i.v. fluids) in the emergency department. Another reason for the discrepancy to the expected prevalence of hypohydration might be, that cognitive dysfunction (MMSE score < 23/30) was one exclusion criterion to ensure that patients could provide informed consent. Cognitive dysfunction is a known risk factor for dehydration [25] due to impaired thirst perception, reduced ability to communicate needs, functional dependence, dysphagia, and polypharmacy [25,26,27,28].

Additionally, decreased vigilance and cognitive impairment are known signs of low-intake dehydration [9,29]. We therefore assume that the actual prevalence of hypohydration, especially low-intake dehydration, was higher in groups of geriatric patients with cognitive impairment, who have not been included in our study protocol. This underlaying condition limits the applicability of our results in patients with impaired condition. In the future our results must be validated in a cohort including patients in diverse cognitive stages to further improve HS-assessment in this heterogenous population.

#### 4.1.1. Laboratory Parameters

Neither serum osmolarity nor serum sodium differed between the hydration groups. In our cohort only four patients met the criteria of a low intake dehydration (Serum sodium > 145 mmol/L and serum osmolarity > 300 mmol/L while having a normal serum glucose). Of these patients, only one was rated as being hypohydrated, since the other three showed signs of heart failure and/or kidney disease. In these cases, the elevated serum osmolarity was interpreted as a temporary effect of the diuretic therapy the patients received. We therefore conclude that our cohort of hypohydrated patients mostly represent hypovolemic patients and cases of isotonic dehydration, so that differences in serum sodium and osmolality are not expected.

#### 4.1.2. Axillary Dryness

Previous research has mainly studied the appearance of a dry axilla as a sign for low-intake dehydration, diagnosed by an elevated serum osmolality [9,30,31]. Most studies reported a high specificity, supporting the use of this method as a criterion to rule out low-intake dehydration. However, our data show both, high specificity (82.8%) and high sensitivity (83.3%). Therefore, we hypothesise that a “dry axilla” may be a useful indicator for overall hypohydration as well.

#### 4.1.3. Inferior Vena Cava Ultrasound

Although IVC sonography is an established method for determining the volume status in intensive care patients, little data are available for the geriatric population. Most importantly, to our knowledge, there is no standardised threshold value for the determination of hypohydration in geriatric patients. In our study a threshold of ≤1.95 cm showed a specificity of 90.9% (95% CI: 62.3; 98.4) and a sensitivity of 50.6% (95% CI: 39.8; 61.4). A maximum measured IVC diameter of >1.95 cm can serve as an exclusion criterion for hypohydration and provides important information that helps clarifying differential diagnoses. Comparing markers of diagnostic accuracy with different cut-off values based on the Jouden Index (Table 3, Section 3.4.1) additionally emphasises the utility of our chosen cut-off as a screening method for overall hypohydration as part of standardised HS assessment.

This cut-off differs from previous findings [12,15]. Orso and colleagues suggested a cut-off value of ≤1.55 cm to identify hypovolemic patients [15]. The major difference to our study protocol is the reference standard, which was applied. Orso and colleagues used BUN:Cr as reference standard to identify dehydration. This parameter has been discussed critically in the past, since it is also influenced by various comorbidities such as protein catabolism, chronic kidney disease and others [32]. Similarly, its diagnostic accuracy to detect hypovolemia has been questioned [33]. In our analysis a cut-off at <1.55 cm reached a sensitivity of 63.64% (95% CI: 35.38; 84.83), limiting its utility of as a screening method to detect overall hypohydration. We conclude that while a cut-off at <1.55 cm might predict dehydration according to Orso et al. [15], a cut-off at <1.95 cm can be applied as a marker for overall hypohydration. Others investigated the IVC collapsibility and inspiratory diameter [12]. However, performing breathing manoeuvres can be difficult in spontaneously breathing patients, so that we aimed to investigate the maximum IVC diameter. Additionally, the cohort of this study also included patients as young as 60 years old, limiting its comparability with a geriatric population as in our cohort [12].

### 4.2. Hyperhydration

In our cohort, 20% of the participants were diagnosed with hyperhydration. The clinical assessment (AR) of each patient also considered signs, such as peripheral oedema. Thus, it can be assumed that the high agreement between the clinical rating (AR) and the final diagnosis (MJ and RW) is largely due to the cases of hyperhydration. In these cases, the clinical assessment, including presence of oedema and other signs, had a decisive influence on the final diagnosis.

#### 4.2.1. Inferior Vena Cava Ultrasound

In our study protocol, four parameters reached an AUC greater than 0.8 in detecting hyperhydration, one of them being the ultrasound examination of the IVC. Other researchers have suggested that measuring IVC collapsibility is the most reliable method for examining volume status [12,15]. As described above, it can be difficult to perform breathing manoeuvres to measure IVC collapsibility in spontaneously breathing patients, especially in the older population. This must be anticipated when searching for feasible methods for assessing the HS in geriatric patients. Therefore, we did not measure the IVC collapsibility but focused on the maximum IVC diameter.

To our knowledge, there is no interdisciplinary accepted threshold for diagnosing hyperhydration in either intensive care or geriatric patients. According to our analysis involving the Jouden Index, a threshold ≥ 2.15 cm reliably detects hyperhydration in both the subxiphoid and transcostal angles. As displayed in Table 4, choosing different cut-offs did not improve diagnostic accuracy, as predicted by the Jouden index. However, this cut-off must be put in context.

Following the American Society of Echocardiology a cut-off ≤ 2.1 cm indicates hypovolemia [34] in non-geriatric patients with heart failure. Furthermore, an age-related decrease in the measurable diameter of the IVC was reported [35]. Considering those previous findings, one can state that a cut-off values in the geriatric population must be expected to be lower than in younger adults. Therefore, a cut-off value of ≥2.15 cm appears to be justifiable to predict hyperhydration in an aged population.

It should be noted, however, that IVC ultrasound has limited value in patients with pulmonary hypertension and/or severe tricuspid regurgitation. These conditions have been reported to affect 5–10% of the elderly population [36].

#### 4.2.2. Body Weight and BMI

Both body weight and BMI also achieved an AUC greater than 0.8 in detecting hyperhydration and both were relevantly higher in the hyperhydrated group. Neuendorff and colleagues found a higher BMI and body weight to be a risk factor for low-intake dehydration [37]. The pathomechanism under discussion in this context involves a diminished storage capacity of body water due to a reduced fat-free mass. This is especially seen in patients with sarcopenic obesity, where fat-free mass is reduced in comparison with the increased total body weight in obese patients [37]. In our analysis, fat-free mass did not achieve an AUC greater than 0.8 and therefore was not further analysed. However, fat-free mass was relevantly lower in the hypohydrated group compared with eu- and hyperhydrated patients. Since BMI was also lower in the hypohydrated group in our cohort compared with non-hypohydrated patients, instead of sarcopenic obesity, sarcopenia and anorexia of ageing must be considered as associated condition in our cohort. However, this finding might be confounded by the limited sample size in the hypohydrated group and needs to be further analysed in the future. The detailed differences in BIA-parameters between the hydration groups are shown in Supplementary Materials Table S3.

In addition to weight monitoring, BIA has been suggested as a screening method for shifts in the HS in multimorbid and aged patients [38]. Furthermore, the ECW/TBW-ratio was examined as an indicator of fluid overload and was associated with an increased mortality in patients receiving continuous renal replacement [39]. Using the ECW/TBW-Ratio in fluid management reduced 28-day-mortality in postoperative ICU patients [40]. In our cohort, none of the BIA-parameters achieved an AUC greater than 0.8 in diagnosing either hypo- or hyperhydration.

### 4.3. Strengths and Limitations

The present study has some limitations. The most important limitation is the incorporate bias, which is inevitable with the chosen clinical reference standard in our study protocol. This might lead to an overestimation of the diagnostic accuracy of each method, since the experts had access to all available information. However, each and every single information is important in the decision making for the experts to find the “true” diagnosis. It should be noted that both raters were involved in previous research on this topic and were aware of earlier findings regarding possible methods for detecting HS in geriatric patients [7]. Since the raters had access to all patient information, including the analysed methods, such as IVC sonography and axillary moisture, some degree of incorporate bias is inevitable. Even though they were not involved in the initial standardised HS assessment, but reviewed its results in an anonymous patient record, it is still debatable whether our work reflects the true diagnostic accuracy or merely the influence of each individual method on the experts’ decision making process. However, in the absence of a reliable gold standard, this approach remains the best practice for distinguishing between different stages of the HS. Thus, this approach appears to be justifiable, especially when considering the STARD 2015 guidelines for reporting diagnostic accuracy [17]. Furthermore, due to their extensive knowledge on the challenges and pitfalls of diagnosing HS in older patients, the two experts are likely to find a more accurate diagnosis than any single method could achieve in this multimorbid and diverse population.

Our exploratory pilot study is a single-centre, prospective observation study. It would be useful to widen the study-protocol in a next step to a multicentred study and closer to the admission of old patients in the emergency department. However, we find that the method of a clinical reference standard is one of the very few possible approaches to study the important issue of HS in old patient.

Also, our study aimed to evaluate the diagnostic accuracy of each method under real-life conditions in this heterogeneous geriatric population. Our cohort represents a broad spectrum of underlaying morbidities, including chronic kidney disease and heart failure, which interfere with the results of some of the methods we performed. This, however, puts the importance of our clinical reference standard in the spotlight, since the two raters had full access to all relevant diagnoses of each patient. Disturbances in water and electrolyte metabolism are among to most frequently seen in this population [2,3]. It is therefore essential to test diagnostic methods under everyday conditions rather than under highly standardised circumstances.

As described in many other multifactorial conditions, the clinical approach to a geriatric patient needs to include a bundle of clinical signs and tests, when it comes to assessing their HS. Our study shows that two indicators, the appearance of a “dry axilla” and the IVC diameter, need to be respected more in the clinical reasoning regarding the assessment of the HS in this population.

To our knowledge, this is the first study to investigate the diagnostic accuracy of different methods for assessing the *overall* HS in geriatric patients by comparing them with an expert panel as clinical reference standard. Particularly the substantial agreement between the two experts suggests that it is possible to reliably identify the current state of the *overall* HS. For the future, our preliminary findings will be validated in a multicentre-prospective setting with a broadened patient spectrum.

We suggest that a diagnostic algorithm for assessing the HS should be based on a clinical reasoning, considering axillary dryness and the IVC diameter. Our work therefore contributes to improving the HS assessment in geriatric patients and consequently their medical care.

## 5. Conclusions

After the final analysis of our data and based on the clinical experience gained from the structured diagnostics in our study, our main conclusions are as follows:

- Integrating the palpation of the axillary skin as part of the routine physical examination not only helps to train the investigators in identifying the underreported clinical sign of a “dry axilla” but also helps to evaluate the overall HS in older patients.
- Performing IVC sonography can be challenging. It is therefore crucial to guide young physicians in this procedure, which would be the first step for improving the assessment of the overall hydration status.

The clinical sign of axillary dryness and the IVC sonography contribute significantly to the determination of the *overall hydration status*, including both volume and hydration status in geriatric patients. None of the other methods showed sufficient diagnostic accuracy. In the absence of a gold standard, a structured clinical consensus provides a feasible and reproducible approach to establish a clinical reference standard. In the future, developing a standardised diagnostic algorithm that includes both methods may improve the HS assessment in a geriatric population.

## Figures and Tables

**Table 1:** Baseline patient characteristics stratified by hydration status. Group differences were calculated using Kruskal–Wallis and chi-square test.

Characteristics n (%)	Total 101 (100)	Hypohydrated 12 (11.9)	Euhydrated 69 (68.3)	Hyperhydrated 20 (19.8)	*p*
Age [years] *	80.1 (±6.97)	81.1 (±6.20)	80.3 (±6.95)	78.8 (±7.62)	0.594 ^†^
Gender [female, n] ***	59.0 (58.40)	10.0 (83.30)	40.0 (58.00)	9.0 (45.00)	0.103 ^‡^
Height [cm] *	167.1 (±8.84)	161.4 (±5.28)	167.4 (±8.33)	169.9 (±10.88)	0.011 ^†^
Weight [kg] *	76.9 (±19.91)	61.7 (±13.68)	74.6 (±17.00)	94.1 (±21.62)	**<0.001** ^†^
BMI [kg/m^2^] *	27.4 (±5.88)	23.6 (±4.92)	26.5 (±5.12)	32.5 (±5.91)	**<0.001** ^†^
MMSE [Points] **	27.0 (±3.0)	26.5 (±5.25)	27.0 (±3.0)	27.0 (±2.75)	0.594 ^†^

* Mean ± standard deviation (SD); ** Median ± interquartile range (IQR); *** Absolute (relative frequency); † Kruskal–Wallis Test; ‡ Pearson chi-square test.

**Table 2:** Laboratory measurements in all patients stratified by hydration status.

Characteristics n (%)	Total 101 (100)	Hypohydrated 12 (11.9)	Euhydrated 69 (68.3)	Hyperhydrated 20 (19.8)	*p*
**Serum Parameter**					
S-Osmolarity [mOsmol/L] *	291.4 (±13.37)	294.8 (±13.71)	291.3 (±11.42)	289.7 (±18.95)	0.773 ^†^
S-Sodium [mmol/L] *	136.3 (±5.87)	137.6 (±7.87)	136.62 (±4.69)	134.15 (±7.79)	0.629 ^†^
S-Creatinine [mg/dL] *	1.22 (±0.87)	1.43 (±1.237)	1.108 (±0.728)	1.455 (±1.014)	0.290 ^†^
S-Urea [mg/dL] *	51.3 (±39.99)	54.9 (±51.35)	46.4 (±32.51)	66.5 (±53.09)	0.539 ^†^
BUN:Cr *	20.3 (±10.11)	19.2 (±7.62)	20.4 (±10.47)	20.7 (±10.56)	0.990 ^†^
S-Potassium [mmol/L] *	4.08 (±0.52)	3.94 (±0.322)	4.08 (±0.503)	4.14 (±0.662)	0.521 ^†^
S-Glucose [mg/dL] *	124.2 (±59.86)	130.8 (±87.16)	122.4 (±55.91)	126.5 (±56.62)	0.600 ^†^
**Urine Parameters**					
Urine color ** [0;6 (±IQR)]	4.00 (3.00)	4.0 (±3.25)	4.0 (±3.75)	5.0 (±3.50)	0.387 ^†^
Specific urine gravity [g/mL(±SD)] *	1.0169 (±0.01314)	1.0164 (±0.00888)	1.0180 (±0.0149)	1.0133 (±0.0066)	0.238 ^†^

* Mean ± Standard Deviation (SD); ** Median ± Inter Quartile Ratio (IQR); † Kruskal–Wallis Test.

**Table 3:** Measures of diagnostic accuracy for axillary dryness and IVC sonography in detecting hypohydration.

Diagnostic Method	Sensitivity (95% CI)	Specificity (95% CI)	PPV (95% CI)	NPV (95% CI)	LR+ (95% CI)	LR− (95% CI)
**Palpation of axillary moisture**						
Dry axilla *^,#^	83.3 (55.2; 95.3)	82.8 (73.5; 89.3)	40.0 (23.4; 59.3)	97.3 (90.7; 99.3)	4.83 (2.86; 8.17)	0.20 (0.06; 0.72)
**Maximum IVC diameter, subxiphoidal angle**						
cut-off < 1.55 cm **	63.64 (35.38; 84.83)	81.01 (71.01; 88.14)	31.82 (16.36; 52.68)	94.12 (85.83; 97.69)	3.35 (1.77; 6.34)	0.45 (0.20; 0.99)
cut-off ≤ 1.95 cm **	90.9 (62.3; 98.4)	50.6 (39.8; 61.4)	20.4 (11.5; 33.6)	97.6 (87.4; 99.6)	1.84 (1.38; 2.46)	0.18 (0.03; 1.18)
cut-off < 2.15 cm **	100.00 (74.12; 100.00)	36.71 (26.93; 47.72)	18.03 (10.38; 29.47)	100.00 (88.30; 100.00)	1.58 (1.34; 1.87)	0.00 (0.00; 0.00)

* n = 99; ** n = 90; # defined by ratings below zero on a range from −2 indicating a “very dry” skin in the axilla and +2 indicating “very moist” skin; PPV: Positive Predictive Value; NPV: Negative Predictive Value; LR+: Positive Likelihood Ratio; LR−: Negative Likelihood Ratio; IVC: Inferior Vena Cava.

**Table 4:** Measures of diagnostic accuracy for IVC sonography in detecting hyperhydration.

Diagnostic Method	Sensitivity (95% CI)	Specificity (95% CI)	PPV (95% CI)	NPV (95% CI)	LR+ (95% CI)	LR− (95% CI)
**Maximum IVC diameter, subxiphoidal angle**						
cut-off ≥ 2.50 cm **	47.37 (27.33; 68.29)	90.14 (81.02; 95.14)	56.25 (33.18; 76.90)	86.49 (76.88; 92.49)	4.80 (2.06; 11.22)	0.58 (0.38; 0.90)
cut-off ≥ 2.15 cm **	73.7 (51.2; 88.2)	78.9 (68.0; 86.8)	48.3 (31.4; 65.6)	91.8 (82.2; 96.5)	3.49 (2.07; 5.89)	0.33 (0.16; 0.71)
cut-off ≥ 1.55 cm **	94.74 (75.36; 99.06)	28.58 (19.32; 40.05)	26.47 (17.45; 38.01)	95.24 (77.33; 99.15)	1.33 (1.11; 1.59)	0.18 (0.02; 1.29)
**Maximum IVC diameter, transcostal angle**						
cut-off ≥ 2.50 cm ***	55.56 (26.66; 81.12)	91.43 (77.62; 97.04)	62.50 (30.57; 86.32)	88.89 (74.68; 95.59)	6.48 (1.90; 22.17)	0.49 (0.23; 1.02)
cut-off ≥ 2.15 cm ***	88.9 (56.5; 98.0)	71.4 (54.9; 83.7)	44.4 (24.6; 66.3)	96.2 (81.1; 99.3)	3.11 (1.76; 5.52)	0.16 (0.02; 1.00)
cut-off ≥ 1.55 cm ***	100.00 (70.08; 100.00)	25.71 (14.16; 42.07)	25.71 (14.16; 42.07)	100.00 (70.08; 100.00)	1.35 (1.11; 1.64)	0.00 (0.00; 0.00)

** n = 90; *** n = 44; PPV: Positive Predictive Value; NPV: Negative Predictive Value; LR+: Positive Likelihood Ratio; LR−: Negative Likelihood Ratio; IVC: Inferior Vena Cava.

## Data Availability

The data presented in this study are available on request from the corresponding author. The data is not publicly available due to ongoing analysis of the study.

## Supplementary Materials

The following supporting information can be downloaded at: https://www.mdpi.com/article/10.3390/nu17193129/s1, Table S1: Exclusion and Inclusion Criteria in patient acquisition; Table S2: AUC of the ROC Analysis of each method in detecting hypo- or hyperhydration, and Table S3: Group differences in BIA-parameters stratified by hydration group.

## Abbreviations

The following abbreviations are used in this manuscript:

HS	Hydration Status
IVC	Inferior vena Cava
BUN:Cr	Blood Urea Nitrogen to Creatinine Ratio
BIA	Bioimpedance Analysis
ECW	Extracellular Water
TBW	Total Body Water
ECW/TBW	Extracellular Water to Total Body Water Ratio
MMSE	Mini-Mental State Examination
SD	Standard Deviation
IQR	Interquartile Range
ROC	Receiver Operating Characteristic
AUC	Area Under the Curve
PPV	Positive Predictive Value
NPV	Negative Predictive Value
LR+	Positive Likelihood Ratio
LR−	Negative Likelihood Ratio
CI	Confidence Interval
BMI	Body Mass Index
BIVA	Bioelectrical Impedance Vector Analysis
FFM	Fat Free Mass
