# Supplementary material for: Hydration Status in Geriatric Patients—Subjective Impression or Objective Parameter? The Hydr-Age-Study
Source: Nutrients. 2025 Sep 30;17(19):3129. doi: 10.3390/nu17193129 (PMC12526359; doi:10.3390/nu17193129)
Supplement: Supplementary file 1 [file nutrients-17-03129-s001.zip › nutrients-3884557-supplementary.pdf]

nutrients

## Supplementary Materials

**Table S1.** Exclusion and Inclusion Criteria in patient acquisition

| Category | Exclusion Criteria | Inclusion Criteria |
|---|---|---|
| Age | <65 years | >65 years with a geriatric patient profile |
| | 65-70 years without any geriatric syndromes or comorbidities | >70 years |
| MMSE | <23 of 30 Points | >23 of 30 Point |
| | | MMSE not evaluable, e.g., language barrier but clear understanding in the studies objective |
| Blood sampling | Admissions blood sampling did not take place within 48h after admission | Sampling was performed within 48h after admission |
| Hydration status assessment | Assessment was not feasible within the same day of the admission blood sampling | Same day assessment as the admissions blood sampling |
| Infection or other patient isolation | Patients were isolated because of an infection (e.g., covid 19) or diarrhea, or similar | |

**Table S2.** AUC of the ROC Analysis of each method in detecting hypo- or hyperhydration

| Method | Hypohydration | Hyperhydration |
|---|---|---|
| **Clinical signs and anthropometric measures** | | |
| Axillary moisture | **0.854** | 0.636 |
| Body weight | 0.763 | **0.802** |
| Body height | 0.759 | 0.588 |
| BMI | 0.691 | **0.802** |
| **IVC sonography** | | |
| IVC max. diameter, transcostal | 0.760 | **0.845** |
| IVC max. diameter (cm), subxiphoidal | **0.834** | **0.849** |
| **Laboratory measurements** | | |
| Serum sodium | 0.551 | 0.590 |
| Serum osmolarity | 0.525 | 0.456 |
| Serum creatinine | 0.532 | 0.614 |
| BUN:Cr | 0.511 | 0.493 |
| Serum urea | 0.518 | 0.582 |
| Serum potassium | 0.601 | 0.526 |
| **Urine parameter** | | |
| Urine color | 0.492 | 0.604 |
| Specific urine gravity | 0.496 | 0.627 |
| **BIA** | | |
| BIA Resistance | 0.720 | 0.702 |
| BIA Reactance | 0.647 | 0.647 |
| Phase angle | 0.514 | 0.557 |
| Body Cell Mass | 0.657 | 0.592 |
| BIVA | 0.669 | 0.682 |
| Total Body Water | 0.791 | 0.749 |
| Extracellular Water | 0.748 | 0.735 |

| | | |
|---|---|---|
| ECW/TBW- ratio | 0.521 | 0.556 |
| Intracellular water | 0.705 | 0.633 |
| Fat free mass | 0.787 | 0.709 |
| Fat mass | 0.717 | 0.779 |
| Dry FFM | 0.670 | 0.573 |

**Table S3.** Group differences in BIA-parameters stratified by hydration group

| **Characteristics n (%)** | **Total 101 (100)** | **Hypohydrated 12 (11.9)** | **Euhydrated 69 (68.3)** | **Hyperhydrated 20 (19.8)** | **p** |
|---|---|---|---|---|---|
| Height* | 167.1 (±8.84) | 161.4 (±5.28) | 167.4 (±8.33) | 169.9 (±10.88) | 0.011† |
| Weight* | 76.9 (±19.91) | 61.7 (±13.68) | 74.6 (±17.00) | 94.1 (±21.62) | **<0.001**† |
| BMI* | 27.37 (±5.88) | 23.6 (±4.92) | 26.5 (±5.12) | 32.50 (±5.91) | **<0.001**† |
| Total Body Water* | 39.23 (±9.86) | 31.56 (±5.86) | 38.01 (±7.19) | 47.47 (±13.62) | **<0.001**† |
| Extracellular Water* | 23.14 (±6.98) | 18.26 (±3.13) | 22.37 (±5.60) | 28.38 (±9.43) | **<0.001†** |
| ECW/TBW* | 0.589 (±0.086) | 0.582 (±0.061) | 0.587 (±0.084) | 0.601 (±0.106) | 0.745† |
| Fatfree Mass* | 49.61 (±11.37) | 41.47 (±7.95) | 48.46 (±8.89) | 57.92 (±15.86) | **<0.001**† |
| Dry Fatfree Mass* | 36.33 (±12.39) | 31.38 (±8.71) | 35.67 (±10.23) | 41.24 (±18.36) | 0.149† |
| Fat Mass* | 27.32 (±12.87) | 18.50 (±7.00) | 26.11 (±11.86) | 36.16 (±13.76) | **<0.001**† |

* Mean ± Standard Deviation (SD); † Kruskal-Wallis-Test
